# Empowering cities globally: Four levers for transformative urban adaptation with nature-based solutions

**DOI:** 10.1073/pnas.2315912121

**Published:** 2025-07-14

**Authors:** Loan Diep, Timon McPhearson

**Affiliations:** ^a^Urban Systems Lab, The New School, New York, NY 10003; ^b^Cary Institute of Ecosystem Studies, Millbrook, NY 12545; ^c^Stockholm Resilience Centre, Stockholm University, Stockholm SE-106 91, Sweden; ^d^Beijer Institute of Ecological Economics, Royal Swedish Academy of Sciences, Stockholm SE-104 05, Sweden

**Keywords:** climate adaptation, nature-based solutions, sustainability transitions, transformative adaptation, enabling environment

## Abstract

The latest report of the Intergovernmental Panel on Climate Change showed that upcoming decades are likely to be accompanied with an increase in climate impacts in urban areas, thereby stressing the need for empowering cities to help them address the challenges ahead by closing the urban adaptation gap. The lock-in systems in which cities are often trapped create resistance to change and leads to missed opportunities to cocreate better futures. Putting nature at the center of urban adaptation agenda is a promising vision that nature-based solutions can help realize if city leaders are adequately supported in accessing the knowledge and resources they need to take action. Despite their proven potential, important barriers to the planning, implementation, and management of nature-based solutions (NbS) for urban climate adaptation persist and need to be urgently addressed. We highlight key barriers related to knowledge gaps and dissemination, policy incentives, and financial autonomy faced by city leaders trying to integrate NbS into policy and planning. In this perspective, we propose four levers of transformative adaptation which build on examples from Europe, the United States, Africa, and Latin America and which have proven successful in supporting cities adopting nature-based adaptation actions, including to 1) produce, assess and share knowledge; 2) adopt incentive-based policies and regulations; 3) facilitate access to multiple sources of funding; and 4) create reflexive monitoring mechanisms. If these steps are taken with the goal of addressing vulnerabilities on the ground, they can unleash the potential of NbS to engage on a path of transformative adaptation.

## 1. The Urban Adaptation Gap and the Role of Nature

Each passing year shatters new climate records, with climate change causing damage through impacts that multiply in frequency, intensity, and duration in all regions of the world. As risks become more systemic, simultaneous, and affect multiple locations across different timescales, they can also lead to cascading and compounding effects where chain reactions amplify the impacts of climate hazards (1). Current institutions and infrastructure systems are not able to cope with the extent of the climate challenge (2). In the year 2023 alone, economic losses were estimated at up to US$380 billion globally (3). Under a mid-range scenario of future income development, global annual damages in 2050 could cost an estimated US$38 trillion, with a range of 19-59 trillion dollars (4). Despite the scale and urgency of the challenge, climate action is lagging behind.

**Fig. 1.**
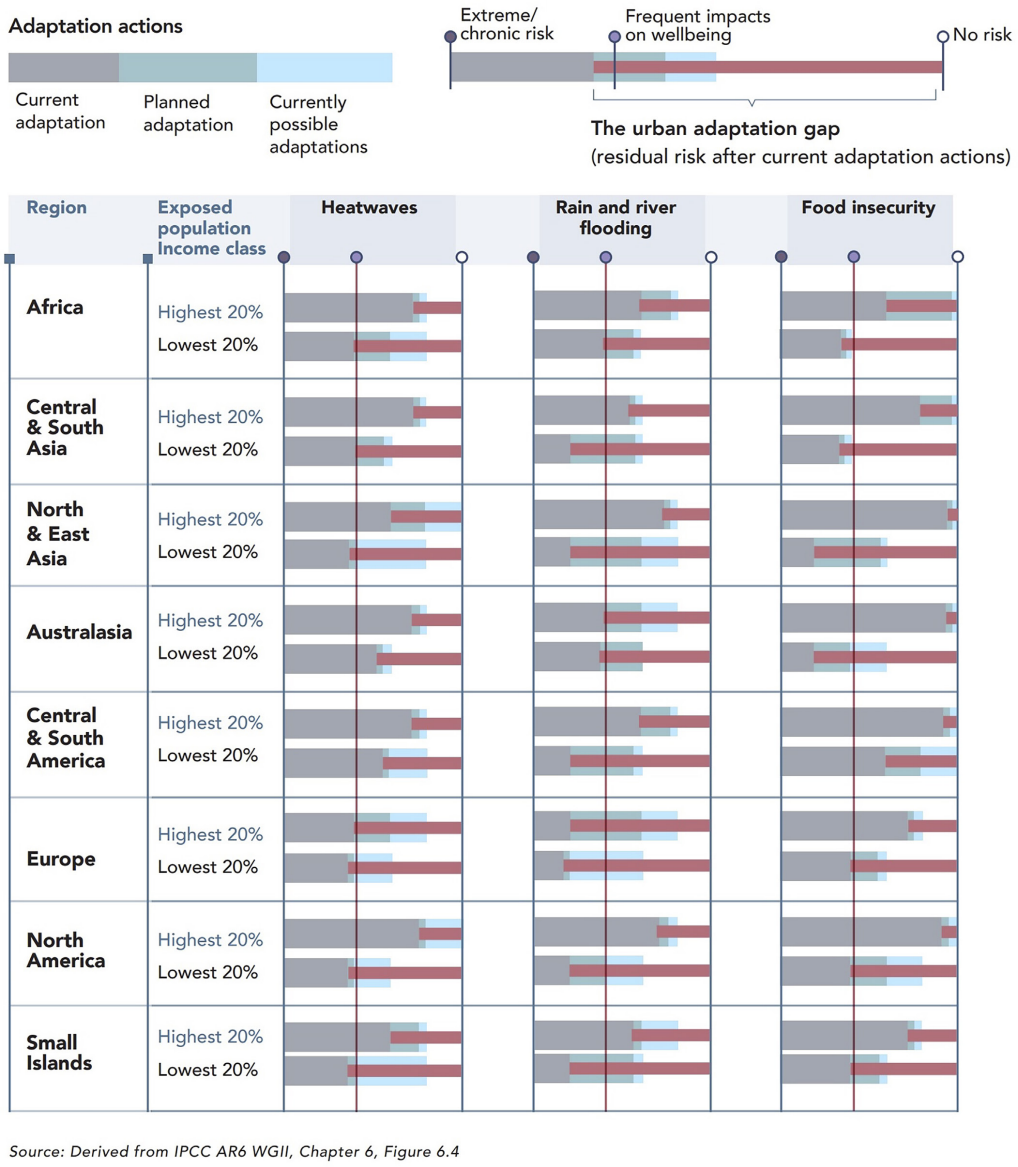
Qualitative assessment of the adaptation gap for urban populations and the adaptation actions needed to reduce risks associated to different climate-related risks. The data are presented for individual world regions and are noncomparative. Within each region, the top and bottom bars compare the poorest and wealthiest population quintiles for adaptation to climate change. Source: Revi et al. (8). Image credit: Reprinted from ref. 8, with permission from Aromar Revi for the Indian Institute for Human Settlements.

Currently, less than 10% of global finance for climate action is directed toward adaptation (5). While mitigation efforts to reduce greenhouse gas emissions remain critical in addressing the root causes of climate change, adaptation strategies are essential to protect populations, ecosystems, and infrastructure from ongoing and future projected climate impacts. This is particularly true in urban settings, where over 55% of the global population resides, where critical infrastructure is concentrated, and where 80% of economic output is produced, making cities fundamental units of climate adaptation as much as mitigation (6, 7). The “Cities, Settlements and Key Infrastructure” Chapter of the latest Assessment Report (AR6) of the Intergovernmental Panel on Climate Change (IPCC), which provides the most comprehensive assessment to date on climate risks and solutions in urban contexts, shows that adaptation efforts are dramatically insufficient in cities (Fig. 1) (1, 8). Increased finance, investment, and local actions that address the adaptation gap must therefore be urgently deployed and focused especially on cities in regions with the most exposed and vulnerable populations.

Climate change adaptation includes adopting measures that help prepare for and respond to the impacts of climate change. A key message across the IPCC is that nature plays a central role in climate adaptation (9). Scientists have been building more and more evidence on the importance of nature-based solutions (NbS) in supporting cities, settlements, and infrastructure to adapt to climate change at multiple scales, with myriad potential cobenefits for health, biodiversity, and equity, thereby supporting societies in facing short-term and long-term risks. The IPCC urban adaptation chapter unpacks how NbS can support climate adaptation in seven ways: through 1) temperature regulation, 2) stormwater regulation and sanitation, 3) coastal flood protection, 4) water provisioning and management, 5) air quality regulation, 6) river flood impact reduction, and 7) food production and security (1).

Considering the role NbS can play among the array of climate adaptation options available, the World Economic Forum estimated the global annual investment opportunity for NbS (i.e., the capital expenditure required) at US$583 billion (10). Investment opportunities in NbS for infrastructure and land-sparing interventions could create more than 59 million jobs in cities worldwide by 2030 (10). Besides, analyses of the business opportunity for NbS showed a potential return of at least US$1.5 trillion in annual business value for the same timeframe. Of course, cities’ actions through NbS and their impact will vary across regions, and many important benefits exist beyond those quantified in economic terms. While considering these, the evidence of the potential of NbS to underpin climate adaptation continues to grow but many cities continue to face major barriers to invest, implement, and maintain them, notably because of knowledge gaps, but also because of political and practical barriers (11). As a result, the mainstreaming of NbS remains low and their potential untapped in many cities and urban regions around the world (12).

In this perspective, we discuss current barriers to mainstreaming NbS and highlight four levers of change for cities to adopt transformative adaption pathways using NbS to help address the urban climate adaptation gap. We do not argue that NbS are silver bullet “fixes” on their own, but rather that they must be central to deeper, systemic changes, particularly through key governance mechanisms within which different actors play a supporting role at multiple levels, from national governments to international funders, nongovernmental organizations, citizens, and scholars.

## 2. Barriers to Nature-Based Urban Adaptation Faced by Local Leaders

### 2.1. Lack of Relevant Knowledge.

Despite fast-growing research on NbS, major knowledge gaps continue to hinder local governments’ actions because decision-makers are faced with limited understanding of ways to optimize the performance of NbS for climate adaptation within their own context (12). This relates to three persisting, interrelated issues: research applicability in practice, NbS practice replicability across contexts, and the wider politics of knowledge production and sharing. First, research knowledge is not always directly applicable by practitioners, including policymakers, city planners, engineers, and designers. High-level policy messages as well as scientific findings require knowledge brokerage institutions to transform this knowledge into relevant, localized forms (13). Deficits in skilled knowledge brokers limit synthesis efforts (14, 15) and create a time lag between scientific research production and its translation into practice, all of which delay dissemination and uptake by decision-makers. However, NbS and climate change are not all about scientific knowledge and require knowledge brokering approaches that focus on innovation and collaboration with diverse knowledge holders (13).

Second, city leaders have limited understanding of what action is replicable in their own context. The current body of NbS knowledge that provides empirical evidence on the impacts of NbS on climate adaptation tends to produce findings that remain context-specific and difficult to reproduce. For example, despite the widespread evidence on NbS strategies for stormwater management, there are still needs to understand what types of green stormwater infrastructure can be effective in reducing flooding impacts and where other solutions will be needed (16). Decision-makers further lack information describing the underlying political context and specific socioeconomic conditions under which NbS have been effective in improving adaptation (and for whom) to be able to apply comparable strategies elsewhere (17). Limited lessons on replicability are also caused by a lack of monitoring and evaluation of NbS’ performance which reflect wider problems of continuity and reflexive approaches in projects (18).

Third, the politics of knowledge production and sharing are an underlying driver to the way NbS knowledge progresses. While Europe has been leading the global production of knowledge on NbS, much less knowledge is available for cities in regions like Africa, the Middle East, and certain parts of Asia (19, 20). Knowledge gaps prevent governments in understudied regions from taking action, all of which skew knowledge production even further, and hamper the understanding of NbS applicability and effectiveness in diverse ecosystems and sociocultural contexts. In relation to urban heat, poor data accessibility has resulted in a lack of cooling solutions serving populations that are already disproportionally exposed to heat burden (21). Even in cities where NbS research is being actively developed, many issues keep emerging because data are not available at the relevant scale (22). We do not argue that the production of NbS knowledge should be solely focused on serving city leaders’ agendas but rather that new ways to produce NbS knowledge are needed. The importance given to the need to make the “business case” for NbS has given priority to the economic valuations of NbS, which reflects wider power dynamics that determine which values and whose values are needed to make decisions and drive investment. This has detrimentally undermined or coopted many other knowledges of, and relationships with nature, notably Indigenous Peoples’ cosmovisions (23). In this regard, urgent attention is needed on different forms of knowledge, and to diverse values of nature to support actions that do not reproduce injustices, but rather push boundaries in ways that contribute to climate justice.

### 2.2. Lack of Financial and Political Incentives.

The high amounts of finance needed for climate adaptation participate in disincentivizing local governments to invest (24). While an investment of US$4.5 to 5.4 trillion is needed every year to build low-emission and climate-resilient urban infrastructure globally, only US$390 billion is actually invested in cities for climate action on average (25, 26). Financial risks keep cities from realizing sustainability ambitions, particularly in the Global South (27). The time needed to see benefits emerge can hamper efforts given the high upfront capital expenditures that can be necessary to implement adaptation measures, including NbS though they can cost 50% less than gray infrastructure alternatives in most instances (10). Operating and maintaining infrastructure is where high costs largely serve as a barrier to the undertaking of NbS-related sustainability transitions. Cities thus struggle to justify large-scale investments, particularly when they face budget constraints and short-term political cycles.

Cities can also struggle to take action on NbS due to the absence of incentives within the international or national policy environment in which they operate. The lack of binding obligations and incoherent policy frameworks refrain cities from adopting adaptation measures. While global climate agreements such as the Paris Agreement set overarching goals, they also lack the mechanisms to enforce mandatory targets at the city level. Where plans exist, they can be weak or outdated. A recent assessment of 167 urban adaptation plans of medium- and large-sized European cities showed that their average score was only one-third of the total possible quality score (28). Low quality scores particularly related to weak participation, monitoring and evaluation aspects, as well as poor plan consistency (28). Since “nature-based visions” are lacking at national levels (29), cities lack incentives to deploy nature-based actions. This absence of accountability can lead cities to adopt a “discretionary” type of action as opposed to an “obligatory” one.

The lack of incentives from the policy and market spheres results in business-as-usual approaches in urban infrastructure development. Reliance on 20th-century modes of development that focus on fail-safe infrastructure continues despite calls for more flexible and “loose-fit” infrastructure designs to deal with uncertainty (30, 31). NbS emerged as “safe-to-fail” and “no-regret” approaches to precisely address this challenge but they need be better prioritized to provide a more adaptable way to manage risks and address future uncertainty (32). Without clear financial benefits or regulatory structures, cities may continue to prioritize other immediate concerns over long-term climate adaptation measures, thereby delaying essential steps needed now.

### 2.3. Limited Autonomy.

Limited autonomy and capacity can hinder action even when leaders have the incentives to take ambitious adaptation measures. Because the lack of political power and access to finance constitutes some of the most significant obstacles faced by cities around the world (33), many of the urban adaptation plans developed in the last decades could not be fully realized. Additionally, urban adaptation plans often lack consistency across their planned measures (28), reflecting a misalignment of risks with measures that are tied to political dynamics. Barriers to implementing plans also include a lack of capacity to manage infrastructure, especially for smaller urban settlements. The poor management of social infrastructure (community facilities, services, and networks) has become particularly problematic despite being an essential part of urban climate adaptation (1).

Many cities find themselves grappling with political constraints that hinder their ability to coordinate climate action (34). Competing priorities across governance levels and sectors pose challenges to the management of interconnected risks. Common climate governance structures consist in power being centralized and not devolved to local governments (35). Additionally, administration turnover on short political cycles keeps long-term planning from happening and hampers investments. Political agendas that prioritize short-term interests over long-term sustainability goals result in policies and regulations that prioritize development projects with immediate economic benefits. City leaders thus find themselves facing resistance from powerful interest groups such as real estate developers and industrial sectors which can undermine efforts to prioritize NbS and comparable urban greening and climate adaptation measures (36).

Between 2010 and 2014, cities received less than 5% of global adaptation finance (26). Multilateral and bilateral donors are too slow or inflexible, resulting in actors on the ground not having access to the financial resources they need, particularly in low- and middle-income countries. These issues have been raised repeatedly in relation to resilience finance, as well as loss and damage finance (37). Even if NbS tend to be less expensive than traditional engineered projects, many types of NbS approaches can be too costly for cities that lack the necessary financial resources to take on large-scale initiatives needed to safeguard their residents. When funding is available, bureaucratic hurdles and changes in political agendas can still slow down or stop implementation. Accessing national climate finance mechanisms can be as complex as accessing international finance for many municipalities. As a result, cities struggle to secure the financing required to take climate action at scale.

## 3. Levers for Transformative Urban Nature-Based Adaptation

The range of barriers that prevent the mainstreaming of urban NbS among local governments are symptomatic of rooted issues of path dependency that remain detrimental to climate action (38). Within the sustainability transition literature, which explores mechanisms that support the transformation of unsustainable socioecological systems, transformative adaptation is a conceptual approach that distinguishes itself from other transition perspectives by calling for more radical forms of systemic change that deeply engage with complex sustainability challenges across scales and address local vulnerabilities (39, 40).

In this part, we focus on four levers for change that have the potential to support cities in moving beyond incremental change and overcome the barriers to nature-based adaptation action. First, produce, assess, and share knowledge relate to the needs to inform approaches that allow experimentation for knowledge production and foster collaboration that facilitates learning exchanges, all of which are essential steps to prompt paradigm shifts from a transformative adaptation perspective (11, 41–43). Second, studies focused on climate adaptation in practice through a transformational lens have identified incentive-based policies and regulations as necessary levers to prompt and guide local initiatives (44, 45). Third, transformative adaptation theories argue for the need to improve city leaders’ access to multiple sources of funding to move beyond business-as-usual approaches and share risks associated with adaptation projects to help them focus on longer-term benefits (44, 46). Fourth, monitoring mechanisms are needed to support reflexive approaches to projects and enable decision-makers to learn from failures, adapt to evolving needs, and share learning, all of which are core governance principles for positive transformation (18, 43, 44). Here, we provide real-world examples to illustrate the application of these levers in the context of nature-based urban adaptation.

### 3.1. Lever 1: Produce, Assess, and Share Knowledge.

Cities are more likely to take action if their decisions are guided by context-relevant data that reduce uncertainty and help them manage risks of failure. By investing in research and development, the European Commission’s Roadmap for NbS set an example of how to map current assets and future needs, a process for which it synthetized existing academic knowledge on NbS, identified theoretical and implementation gaps, and found opportunities for innovation (47). Similarly in the United States, the recently launched National Nature Assessment will be taking stock of the current state of lands, water, wildlife, and the cobenefits they provide considering diverse values of nature, identify areas for NbS implementation, and thereby help implement the country’s own NbS Roadmap (48). Other countries and regions could and should reproduce these initiatives at different governance levels through multi-actor collaborations designed to address current knowledge biases and thereby create a solid knowledge base for nature-based climate adaptation strategies and actions at the local level.

However, such assessments will not be enough to facilitate rapid action from city leaders who need access to relevant information that can be directly translated into tangible action. International city networks like ICLEI - Local Governments for Sustainability, the Climate Alliance and C40 provide structures that allow local governments to directly tap into the wealth of collective intelligence and share data and knowledge at city-level, for example, through online resources, training programs and workshops on new technologies or effective policies (49). The structures in which they operate facilitate horizontal collaboration and help cities build their capacity to shape and implement innovative strategies outside of national to local top–down structures. Notable examples of cities supported by ICLEI in their nature-based strategies include Campinas (Brazil) which has championed the implementation of an urban biodiversity agenda in Latin America (50). In East Africa, ICLEI’s Urban Natural Assets for Africa program has supported the cities such as Kisumu (Kenya) and Quelimane (Mozambique) where NbS could be implemented at community level (51). Other networks such as professional associations, academic extension agencies, and committees that develop technical standards also play a significant knowledge brokering role by linking research and practice with context-specific relevance.

### 3.2. Lever 2: Adopt Incentive-Based Policies and Regulations.

Cities are more likely to design and implement their own plan of action where they are guided by a vision and accompanying goals or targets as well as an incentive-based policy environment. Policies for urban adaptation must consider multiscale benefits and support trade-off assessments (7). Local Agenda 21 and the Convention on Biological Diversity (CBD) set global agendas for countries and cities to have sustainable development strategies. Building on the CBD, the European Union 2020 Biodiversity Strategy established specific goals for member states and offered various programs to support urban greening and biodiversity initiatives. These influenced strategies in various European countries. In Portugal for example, the national strategy informed Lisbon’s Biodiversity Strategy which, based on a set of key indicators, took a number of implementation actions, notably through a network of green infrastructure helping the city counteract the urban heat island effect (52).

Salka et al’s analysis of the “enabling environment” for BioCities highlights the strength of mission-oriented policies in empowering emergent solutions (53). The Global Biodiversity Framework (GBF) aims at setting the new stage for coordinated action for biodiversity conservation and ecosystem restoration. The GBF outlines ambitious goals such as the 30×30 target which calls for 30% of the earth’s land and sea to be conserved (54). Every country will enforce targets internally differently, but this could be particularly challenging for highly urbanized countries. To best achieve targets while ensuring policy coherence, frameworks like the GBF must be accompanied with technical and financing mechanisms to enable countries and cities to leverage funding opportunities and develop and implement NbS strategies tailored to their specific needs and vulnerabilities. Significant transformations will require clear incentives to bridge the gap between initial investments, management, and maintenance costs, and long-term benefits.

Incentive-based policies and regulations can be powerful tools that can take various forms ranging from grant frameworks, to tax breaks, low-income loans, and payment for ecosystem services programs (44, 45). Lessons from nature-based adaptation initiatives in the agricultural sector show that the introduction of new financial incentives of regulations can support actors who are already willing to implement NbS, however, these instruments might not support those resistant to implement NbS. In such cases, additional facilitating levers such as activities centered on knowledge-sharing and awareness might be necessary (45).

### 3.3. Lever 3: Facilitate Access to Multiple Sources of Funding.

To make NbS a central component of urban climate adaptation strategies, city leaders require access to financial instruments that have a stronger focus on climate adaptation and allow for more investment in small-scale, connected green infrastructure (as opposed to monofunctional, large-scale, gray infrastructure) as well as non-infrastructural climate actions (e.g., community savings) (55, 56). Those instruments must come from multiple sources and range from grants to concessional loans and private sector finance in order to give cities flexibility as they invest in technologies and infrastructure, workforce training, and ongoing maintenance. Policies that promote innovative partnerships can unlock new sources of funding and thereby leverage resources as long as they enable capacity-building on the ground and focus on long-term sustainability.

Freetown in Sierra Leone represents a strong example of mobilization of blended funding for climate action. In 2020, the city launched the “Freetown the Treetown” campaign which has consisted in reforesting the city to build resilience against climate hazards including flooding and landslides. Led by the Freetown City Council and supported by the national government, the campaign has been co-financed by international partners such as the Environmental Foundation for Africa and the World Bank. It has been implemented with a network of community-based organizations, where city residents have received financial compensation to plant, nurture and monitor trees. By the end of 2023, the initiative had led to over 977,000 planted trees, with a survival rate of 82% owing to the monitoring strategy supported by local stewards (57). Freetown has further created a financing platform by tokenizing trees to leverage investment from the private sector using carbon credits and thereby secured a self-sustaining model. This initiative has played a pivotal role in enabling further support to implement the “Freetown Climate Action Strategy” for 2022–2030, which represents the city’s attempt toward delivering on its commitment to the Paris Agreement (58).

Opening up diverse financial pathways, together with the creation of a supportive policy environment will not only foster city engagement but also create the foundation to mobilize the financial and nonfinancial resources (e.g., expertise) needed to take action. The case of Freetown shows that when local governments have the power to enact their own strategies, it can encourage public and private sector stakeholders to further invest in initiatives. These investments have led to positive ripple effects, such as workforce development that offers related career pathways, job training, and local job creation that improves financial security (57). Through proactive leadership and the inclusion of its residents, Freetown has emerged as a pioneering city when taking the lead in managing its climate action plan, and thereby, setting an example for other cities.

### 3.4. Lever 4: Create Reflexive Monitoring Mechanisms.

Reflexive monitoring systems do not only enable progress to be tracked through time and evaluate NbS performance through the use of indicators but also allow the integration of findings into NbS design and implementation through iterative processes. Though the International Union for Conservation of Nature’s “Global standards for Nature-Based Solutions” provides a starting point (59), more frameworks are needed to understand NbS monitoring under various contexts. Since NbS can take multiple forms and measuring multifunctionality is one of the most complex aspects of NbS monitoring, a combination of tools, methods, and data are required for the development of comprehensive, but easily implementable frameworks. For example, Kumar et al. (60) argue that to monitor the performance of NbS against five types of risks (floods, droughts, heatwaves, landslides, storm surges and coastal erosion), a mix of ground-based measurements and remote sensing data can be applied to support the evaluation of existing uncertainty. However, these require important resources including equipment, time, and budget.

The most effective use of external investments into monitoring may be to support approaches developed with or by communities themselves. Citizen science initiatives for environmental monitoring have been widely successful over the past decade, notably with the growing use of smartphone applications (e.g., CoastSnap to help monitor coastal erosion) (61). These can be a valuable approach for continuity in data collection through the lens of local experts, as long as mechanisms do not fall into tokenistic approaches or other instrumental forms of participation. Wolff et al. (62) demonstrate through experiences of monitoring floods in collaboration with communities in Fiji and Indonesia that citizen science can significantly advance risk management practices while highlighting that new responsibilities need to be negotiated with participants. By confronting business-as-usual approaches and changing power dynamics, participatory forms of monitoring approaches can play a key role in supporting transformative change through NbS.

## 4. Realizing the Potential of NbS by Transforming Cities

The accelerating impacts of climate change and the likelihood that the upcoming decades will see higher impacts on cities stress the magnitude of a challenge that requires fundamental changes. With barriers persisting, NbS will be implemented within the same structures that have been recreating vulnerability, without harnessing their transformative potential. However, fostering an enabling environment for transformative adaptation using the full potential of NbS can help cities adapt to climate change in ways that protect their residents, economies, and critical infrastructure and ecosystems across scales. The proposed key levers of change acknowledge the intricate interplay between global environmental challenges and local dynamics. NbS experiences remind us that optimizing NbS’ performance ultimately depends on contextual socio-institutional factors that relate to political jurisdiction, administrative structures, sectoral interests and decision timeframes, and more importantly, to power dynamics that influence what values matter in decision-making. National governments, scientists, intermediary organizations, the private sector, and citizens need to work together to support cities leaders in engaging on a path of transformation and craft innovative and impactful NbS strategies that will help close the urban adaptation gap.

## Data Availability

All study data are included in the main text.
